# Improving child health care in Botswana: what can be done?

**DOI:** 10.11604/pamj.2021.39.242.29139

**Published:** 2021-08-16

**Authors:** Lynn Tuisiree Tjirare, Lebapotswe Tlale

**Affiliations:** 1 University of Botswana, Gaborone, Botswana,; 2 Ministry of Health and Wellness, Gaborone, Botswana

**Keywords:** Child health, Botswana, integrated management, childhood illnesses

## Abstract

Access to appropriate healthcare for children remains a challenge in Botswana, as evidenced by the under five mortality rate and integrated management of childhood illness indicators. Successful implementation of the integrated management of childhood illnesses strategy can drastically reduce child mortality through innovation, national health care worker training coverage, enhanced supervision and use of guidelines.

## Opinion

Child health is a significant aspect of life that cannot be over-emphasized. An unwell child has poor growth, community integration, poor cognitive development and ultimately poor economic participation in adulthood [1,2]. It is therefore imperative that all countries invest heavily in ensuring the well-being of a child. Access to appropriate healthcare for children remains a challenge in Botswana according to my experience and as evidenced by the under-five mortality rate and integrated management of childhood illness (IMCI) indicators [3]. The Ministry of Health and Wellness (MOHW) is the custodian of IMCI implementation through the division of child health in Botswana. IMCI aims to reduce under five mortality and promote growth and development. It was introduced in 1997 in Botswana. Although Botswana is reported as a country that has incorporated the IMCI strategy into the national strategy [4], there is no document that explicitly describes the IMCI strategy in Botswana and the pathway of achievement. Rather within the MOHW strategic plan, IMCI implementation is one of the ways that is put forth as conduit to decreasing child mortality. At present, Botswana is said to have more than 90% of districts implementing IMCI and 50-74% of facilities having at least 60% of healthcare worker (HCW) trained in IMCI [4].

Despite the training achievements and presence of regional coordinators, the impact is somewhat lacking. This is evidenced by the poor performance of indicators reflected in the IMCI health facility 2017 survey. This survey showed that diarrhea and pneumonia were the leading causes of mortality and were poorly managed. Only 3% of children with diarrhea were correctly managed while 43% of children with pneumonia were correctly treated. Furthermore, only 17% of children were assessed for danger signs [5]. The above, depicts the poor implementation of IMCI in health facilities. Numerous researches on the barriers to IMCI implementation have been done globally as well as in Botswana. The key challenges that emerge are: 1) insufficient financial resources; 2) lack of training, mentoring and supervision; 3) length of time required for effective IMCI consultations; 4) client expectation [6,7]. In order to improve child health care in Botswana the following was recommended:

**Strategic planning:** the IMCI program should develop a national strategic document and an operational plan document. This would allow for a comprehensive and easy review and monitoring of the program achievements and challenges.

**Training:** all health care workers who provide service to children should be trained in IMCI. Therefore, trainings should continue, to achieve full coverage of all healthcare workers especially for those in primary care. This will also address issues of distributive equity of trained HCW.

**Supervision:** since training alone does not guarantee implementation of guidelines, other measures to enforce implementation should be put in place. It is evident that the supervision capacity or strategy of the regional coordinators is not achieving the desired effect as even the trained HCW are not utilizing the IMCI guidelines [5]. An IMCI coordinator situated at the District Health Management Teams (DHMT) may enhance implementation of IMCI by timely training of all staff and supervising HCW to use IMCI guidelines during all child consultations. The proposed DHMT IMCI coordinator would facilitate under five child cards clinical auditing to assess adherence to the IMCI processes within health facilities.

**Comprehensive child welfare clinics:** the already existing child welfare clinics (CWC) can be well-rounded with addition of child consultation by IMCI trained nurses and doctors. Currently CWC focuses on nutrition monitoring only but a comprehensive child welfare clinic would better improve the health of our children through integration.

**Community education:** intensive and focused health education of caregivers on what to expect when their child is consulted appropriately via IMCI should be done. An educated client, who knows what service to expect, will hopefully force the health care worker to render the appropriate service to children. Successful implementation of the IMCI strategy across the country can drastically reduce child mortality. Botswana will be well on its way to achieve the sustainable development goal 3 once the challenges are addressed effectively and in a timely manner. National HCW training coverage, supervision and ensuring use of guidelines as well as comprehensive childcare clinics are key to efficacious IMCI implementation.
